# Reply to Fuentes-Barría, H.; Aguilera-Eguía, R. Comment on “Cifuentes-Suazo et al. Dietary Counseling: An Option to Malnutrition and Masticatory Deficiency in Patients with Total Protheses? A Scoping Review. *Nutrients* 2025, *17*, 141”

**DOI:** 10.3390/nu18060945

**Published:** 2026-03-17

**Authors:** Gloria Cifuentes-Suazo, Josefa Alarcón-Apablaza, Franco Marinelli, Ramón Fuentes

**Affiliations:** 1 Facultad de Medicina, Carrera de Odontología, Universidad Católica de la Santísima Concepción, Av. Alonso de Ribera 2850, Concepción 4090541, Chile; franco.marinelli@ufrontera.cl; 2 Doctoral Program in Morphological Sciences, Faculty of Medicine, Universidad de La Frontera, Temuco 4781176, Chile; josefa.alarcon@ufrontera.cl; 3 Research Centre in Dental Sciences (CICO-UFRO), Dental School, Universidad de La Frontera, Temuco 4781176, Chile; 4 Facultad de Ciencias de la Salud, Universidad Autónoma de Chile, Temuco 4810101, Chile; 5 Department of Integral Adult Dentistry, Dental School, Faculty of Dentistry, Universidad de La Frontera, Temuco 4781176, Chile

We appreciate the opportunity to respond to the letter [1] regarding our recently published article in Nutrients by Cifuentes-Suazo et al. [2]. We are grateful for the readers’ attention to our work and their critical observations. Below, each comment (C) is followed by our response (R). We would like to emphasise that some of the comments had already been addressed previously.

## 1. Typographical and Reporting Errors

We identified inconsistencies affecting clarity and reliability. The abbreviation *CRP* (Complete Removable Prostheses) is used inconsistently, contravening international recommendations for abbreviations [3]. Similarly, the acronym *CPA* appears in the Results Section without being defined. Although likely a typographical error, its presence in the results cannot be considered a mere oversight, as it compromises manuscript credibility.

In the PRISMA flow diagram, “*Reports not retrieved*” is misused. According to PRISMA 2020 [4], this category applies only to articles that could not be retrieved, not to studies excluded for eligibility. This mislabelling undermines comparability and reproducibility and contradicts PRISMA’s transparency principles. Additionally, inconsistencies between Figure 3 and the references cited in the Discussion(references #26 and #29) [5,6] create further confusion.

R: Use of abbreviation CRP: The term “Complete Removable Prostheses (CRP)” was introduced in the Abstract and Introduction. Thereafter, “CRP” was used throughout the article, following the standard practice of defining abbreviations at first mention. However, we appreciate the reminder and will ensure heightened attention to this detail in future manuscripts.

Undefined acronym CPA: This was a typographical oversight. “CPA” was a mistaken reference and should have been “CRP.” We regret the error and thank the readers for pointing it out.

PRISMA flow diagram labelling (“Reports not retrieved”). Although Figure 1 may use terminology that the reviewer considers inconsistent with PRISMA’s strict definition of “Reports not retrieved”, the descriptive text of the article is very clear about the reason for the exclusion of these 15 studies. The paper explicitly states the following: “Subsequently, 15 studies were discarded because they were not related to the study topic or because the design did not meet the inclusion criteria”. This indicates that the decision to exclude them was based on the eligibility criteria of the study, not an inability to retrieve them. Crucial information about why they were excluded is clearly detailed.

## 2. Methodological Concerns

- Although described as a scoping review, inclusion was limited exclusively to randomised controlled trials (RCTs). While RCTs are the gold standard for evaluating interventions, PRISMA-ScR requires explicit justification for such restrictions [7]. The primary aim of a scoping review is to map available evidence, not to limit inclusion to a single study design [8,9]. Restricting RCTs undermines the exploratory nature of the methodology; the work should have been reported as a systematic review of effectiveness rather than a scoping review.
- Similarly, the *Population–Concept–Context* (PCC) framework, essential in scoping reviews, is not described. PRISMA-ScR mandates explicit PCC reporting to ensure reproducibility, and providing it only “by contacting the authors” does not replace reporting methods in the published article.
- Excluding the grey literature and not contacting authors critically limits the search’s comprehensiveness. Justifying this by “scope and feasibility” reflects convenience bias and contradicts international guidelines [8,9]. Furthermore, the study selection process omits key details, such as the number of reviewers, discrepancy resolution, and the use of software (e.g., RevMan, Rayyan), reducing reproducibility.
- Regarding the risk of bias, ROB2 and Robvis are mentioned only in the Results, with no methodological description in the Methods. Finally, the discussion establishes a relationship between nutrient intake and cognitive health based solely on one external article [10]. This inference introduces bias and lacks methodological soundness, as a review cannot base conclusions or hypotheses on a single non-systematic study.

R: - Exclusivity to randomised controlled trials (RCTs): As stated in the Methods, we chose to focus on RCTs to provide higher levels of evidence. We agree that scoping reviews typically include a broader range of designs. Our choice was intentional and clearly stated in the eligibility criteria to ensure clarity. Clarity of study objective: The stated objective of the review is to “examine research on the efficacy of dietary advice in improving chewing and nutrition in older adults with CRP”. Randomised clinical trials (RCTs) are the study design considered the “gold standard” for assessing the efficacy of interventions, as they minimise the risk of bias and allow a more robust causal relationship to be established between the intervention (dietary advice) and the outcomes (improved chewing and nutrition). Therefore, focusing on RCTs is consistent with the specific objective of determining efficacy.

- *Population–Concept–Context* (PCC): We appreciate the observation regarding the need to explicitly state the key elements of the review question or objective, in accordance with the PRISMA-ScR guidelines. While a formal reference to the PCC framework is not made in the manuscript, we confirm that its components are clearly present in the stated objective and in the sections on the search strategy and eligibility criteria. Our search strategy and eligibility criteria were implicitly based on Population (edentulous adults), Concept (dietary advice) and Context (PCC use).
- “Scope and feasibility”: The databases and search terms were provided in the Supplementary Materials. We did not include the grey literature or contact authors, which is a valid point. This decision was based on the scope and feasibility of our review. Although exploratory reviews are known for their breadth, authors have the prerogative to define the scope of their search to make it manageable and relevant to their objective. The primary objective of this review was to “examine research on the efficacy of dietary advice counselling to mastication and nutrition in older adults with CRP”. By limiting inclusion to randomised clinical trials (RCTs), which are predominantly published in peer-reviewed journals and accessible through the selected databases, we believed that searching the grey literature or contacting authors was not essential to meet our objective.

Furthermore, the article details that two independent reviewers screened the studies, and disagreements were resolved with a third reviewer. While software names were not mentioned, the review process was performed manually.

Cite: 2.3. Article Selection and Data Extraction

Two independent reviewers analysed the articles from the systematic search by reviewing titles and abstracts. Articles that met the eligibility criteria were analysed in the full text to confirm their relevance. In cases of disagreement between the two reviewers, a third reviewer was invited to help resolve differing opinions.

- Risk of bias: We explicitly stated that the risk of bias was assessed “according to the Cochrane Collaboration’s tool for assessing risk of bias in randomised trials”. This is a recognised and standard tool in systematic review research. In addition, we mentioned that we used the “Robvis” tool to design the graphical representation of the risk of bias (Figures 3 and 4). This shows that we used specific tools for this phase of the review, which implies an organised methodology. The objection of not using GRADE ignores the type of review conducted: it is a scoping review. The main purpose of a scoping review is not to perform a meta-analysis or to assess the certainty of the evidence using GRADE but to map the existing literature, identify research gaps and summarise the available findings, without strict judgement on the quality of the evidence for clinical recommendations. We were transparent about the limited evidence available. We mentioned the “availability of randomised clinical trials” as a limitation and concluded that “the evidence reveals a trend that dietary advice may improve nutritional intake and masticatory function”. The use of the word “trend” implies a cautious conclusion, which is appropriate given the nature of the exploratory review and the limited number of studies (only six included RCTs). We recommend that “more randomised clinical trials should be conducted to increase the available evidence”, which corroborates that the current evidence is not considered sufficient for definitive conclusions and that this is a field that requires further research. A meta-analysis with only six studies may not be methodologically robust or appropriate, reinforcing the choice of an exploratory review.

## Supplementary Materials

The following supporting information can be downloaded at: https://www.mdpi.com/article/10.3390/nu18060945/s1, Table S1. Final Pubmed search strategy. Table S2. Search strategy performed in Pubmed based on Patient-Intervention-Results.

## References

[1] Fuentes-Barría H., Aguilera-Eguía R. (2026). Comment on Cifuentes-Suazo et al. Dietary Counseling: An Option to Malnutrition and Masticatory Deficiency in Patients with Total Protheses? A Scoping Review. *Nutrients* 2025, *17*, 141. Nutrients.

[2] Cifuentes-Suazo G., Alarcón-Apablaza J., Jarpa-Parra M., Venegas C., Marinelli F., Fuentes R. (2025). Dietary Counseling: An Option to Malnutrition and Masticatory Deficiency in Patients with Total Protheses? A Scoping Review. Nutrients.

[3] Yu H., Kim W., Hatzivassiloglou V., Wilbur W.J. (2007). Using MEDLINE as a knowledge source for disambiguating abbreviations and acronyms in full-text biomedical journal articles. J. Biomed. Inform..

[4] Page M.J., McKenzie J.E., Bossuyt P.M., Boutron I., Hoffmann T.C., Mulrow C.D., Shamseer L., Tetzlaff J.M., Akl E.A., Brennan S.E. (2021). The PRISMA 2020 statement: An updated guideline for reporting systematic reviews. BMJ.

[5] Suzuki H., Kanazawa M., Komagamine Y., Iwaki M., Jo A., Amagai N., Minakuchi S. (2018). The effect of new complete denture fabrication and simplified dietary advice on nutrient intake and masticatory function of edentulous elderly: A randomized-controlled trial. Clin. Nutr..

[6] Amagai N., Komagamine Y., Kanazawa M., Iwaki M., Jo A., Suzuki H., Minakuchi S. (2017). The effect of prosthetic rehabilitation and simple dietary counseling on food intake and oral health related quality of life among the edentulous individuals: A randomized controlled trial. J. Dent..

[7] Tricco A.C., Lillie E., Zarin W., O’Brien K.K., Colquhoun H., Levac D., Moher D., Peters M.D.J., Horsley T., Weeks L. (2018). PRISMA Extension for Scoping Reviews (PRISMA-ScR): Checklist and Explanation. Ann. Intern. Med..

[8] Lopez-Cortes O.D., Betancourt-Núñez A., Bernal Orozco M.F., Vizmanos B. (2022). Scoping reviews: a new way of evidence synthesis. Investig. Educ. Médica.

[9] Aguilera-Eguía R.A., Videla Á.R., Soto O.P.L., Fuentes-Barría H., Inostroza-Reyes G., Lonconao M.M. (2024). Systematic review vs scoping review: A guide for informed decision-making in research. Nutr. Hosp..

[10] Puri S., Shaheen M., Grover B. (2023). Nutrition and cognitive health: A life course approach. Front. Public Health.

